# Outcome of laparoscopic paraesophageal hernia repair in octogenarians: a registry-based, propensity score-matched comparison of 360 patients

**DOI:** 10.1007/s00464-018-06619-4

**Published:** 2018-12-10

**Authors:** Ralph F. Staerkle, Ilan Rosenblum, Ferdinand Köckerling, Daniela Adolf, Reinhard Bittner, Philipp Kirchhoff, Frank S. Lehmann, Henry Hoffmann, Philippe M. Glauser

**Affiliations:** 1 Department of General and Visceral Surgery, University Hospital Basel, Spitalstrasse 21, 4031 Basel, Switzerland; 2 Department of Surgery and Center for Minimally Invasive Surgery, Academic Teaching Hospital of Charité Medical School, Vivantes Hospital, Neue Bergstrasse 6, 13585 Berlin, Germany; 3 StatConsult GmbH, Halberstädter Straße 40 a, 39112 Magdeburg, Germany; 4 Winghofer Medicum Hernia Center, Winghofer Straße 42, 72108 Rottenburg am Neckar, Germany; 5 Division of Gastroenterology and Hepatology, University Hospital Basel, Petersgraben 4, 4031 Basel, Switzerland

**Keywords:** Paraesophageal hernia repair, Complications, Elderly patients, Propensity score-based, Matched-pair analysis

## Abstract

**Background:**

Paraesophageal hernias (PEH) tend to occur in elderly patients and the assumed higher morbidity of PEH repair may dissuade clinicians from seeking a surgical solution. On the other hand, the mortality rate for emergency repairs shows a sevenfold increase compared to elective repairs. This analysis evaluates the complication rates after elective PEH repair in patients 80 years and older in comparison with younger patients.

**Methods:**

In total, 3209 patients with PEH were recorded in the Herniamed Registry between September 1, 2009 and January 5, 2018. Using propensity score matching, 360 matched pairs were formed for comparative analysis of general, intraoperative, and postoperative complication rates in both groups.

**Results:**

Our analysis revealed a disadvantage in general complications (6.7% vs. 14.2%; *p* = 0.002) for patients ≥ 80 years old. No significant differences were found between the two groups for intraoperative (4.7% vs. 5.8%, *p* = 0.627) and postoperative complications (2.2% vs. 2.8%, *p* = 0.815) or for complication-related reoperations (1.7% vs. 2.2%, *p* = 0.791).

**Conclusions:**

Despite a higher risk of general complications, PEH repair in octogenarians is not in itself associated with increased rates of intraoperative and postoperative complications or associated reoperations. Therefore, PEH repair can be safely offered to elderly patients with symptomatic PEH, if general medical risk factors are controlled.

**Electronic supplementary material:**

The online version of this article (10.1007/s00464-018-06619-4) contains supplementary material, which is available to authorized users.

Hiatal hernias are divided into types I–IV, of which approximately 5–15% are paraesophageal hernias (PEH) (types II–IV) [1]. PEH is defined as herniation of the stomach and/or other viscera through a dilated hiatal aperture alongside the esophagus [1, 2]. These hernias tend to be found more frequently in elderly women, although adults of any sex and age may be affected [3]. Dysphagia, vomiting, and regurgitation, often associated with retrosternal pain, are typical symptoms [3]. Pharmacological treatment is often unsatisfactory since PEH symptoms are mostly related to the mechanical effects of the hernia.

The annual incidence of acute symptoms in patients with PEH ranges between 0.7 and 7% [4, 5]. Emergency repairs of PEH are associated with a sevenfold increase in mortality compared with elective repairs [6]. Several studies showed that elective laparoscopic PEH repair has a low morbidity resulting in significantly improved quality of life [3, 7–11]. Although elective PEH repair may be used increasingly in older patients [12], the assumed higher perioperative morbidity in elderly patients may dissuade clinicians from seeking a surgical solution.

However, data on perioperative outcomes of elective PEH repair in octogenarians or older patients are sparse. One study analyzing short-term outcomes associated with PEH repair in patients aged 80 years and older revealed higher rates of minor morbidity, but no significant differences in mortality or major morbidity rates compared to younger patients [11].

In this registry-based, matched-pair analysis, intraoperative, postoperative, and general complication rates after elective PEH repair in patients ≥ 80 years were assessed and compared to younger patients.

## Methods

The Herniamed Registry is a multicenter, internet-based hernia registry [13] with 644 participating hospitals and surgeons in private practice (Herniamed Study Group) in Germany, Austria, and Switzerland (status: January 5, 2018) who have shared data on their patients undergoing routine hernia surgery. All patients signed an informed consent form agreeing to participate. As part of the information provided to patients regarding participation in the Herniamed Quality Assurance Study and signing the informed consent declaration, all patients were informed that the treating hospital or medical practice should be informed about any problem occurring after the operation and that the patient should have a clinical examination if needed. All postoperative complications occurring up to 30 days after surgery are recorded.

**Fig. 1:**
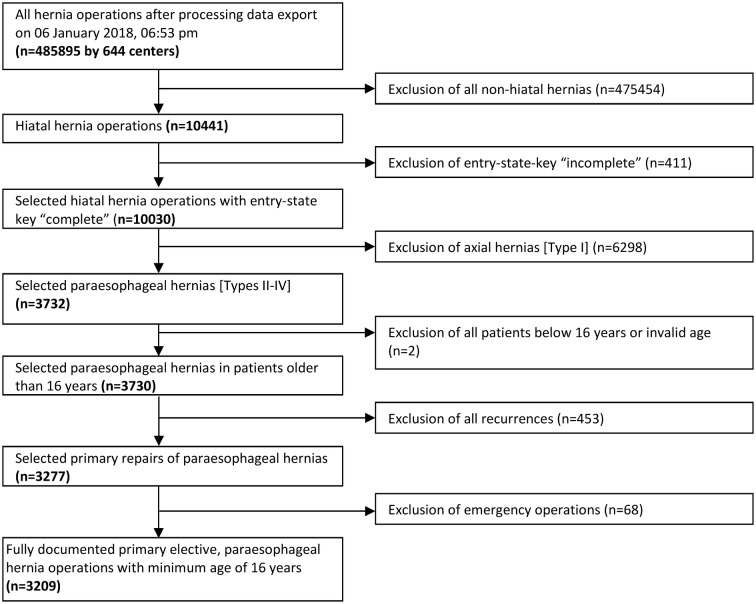
Flowchart of patient inclusion

The current analysis compares the prospective data gathered on PEH repairs in octogenarians (≥ 80 years) and younger patients (< 80 years) between September 1, 2009 and January 5, 2018 using a matched-pair analysis. The main inclusion criteria were hiatal hernia operation, complete entry state, paraesophageal hernia (types II–IV), minimum age of 16 years, primary operation, and no emergency repair. In total, 3209 patients were enrolled (Fig. 1). Pairwise propensity score (PS) matching analysis was performed for these 3209 patients to obtain homogeneous comparison groups.

The data collected were age, body mass index (BMI), type of fundoplication, type of hiatal hernia, type of hiatal repair, American Society of Anesthesiologist (ASA) status, and gender.

The following risk factors were assessed as possible risk factors for an adverse outcome: chronic obstructive pulmonary disease (COPD), diabetes mellitus, aortic aneurysm, immunosuppression, steroids, smoking, coagulation disorder, or antiplatelet or anticoagulant therapy. All analyses were performed with the software SAS 9.4 (SAS Institute Inc., Cary, NC, USA) and intentionally calculated to a full significance level of 5%, with the exception of post hoc analyses for single general complications. Here, adjustment for multiple testing was made using a Bonferroni correction (factor 16).

Analogous to previous registry-based analyses [14], intraoperative complications (bleeding, injury to esophagus, bowel, spleen, stomach, or liver), postoperative complications (esophageal perforation, gastric perforation, bleeding, infection, wound healing disorder, or ileus), overall complications, and complication-related reoperations were compared between age groups using, first of all, PS matching. Matched samples were analyzed with McNemar’s test. Outcomes are given as the non-diagonal elements of the 2 × 2 frequency table, which represent differences in the matched samples, the corresponding *p*-values, and the odds ratio (OR) estimates for matched samples. PS matching was performed using greedy algorithm and a caliper of 0.1 standard deviations. The variables used for matching were sex (male/female), type of fundoplication, BMI (kg/m^2^), hernia type (II, III, IV), risk factors (COPD, diabetes, aortic aneurysm, immunosuppression, steroids, smoking, coagulation disorder, anticoagulants, antiplatelet therapy), and ASA classification (I, II, III, IV). The balance of the matched sample was checked using standardized differences (also given for the original sample) that should not exceed 10% (< 0.1) after creating matched pairs. For pairwise comparison of matching parameters between age groups (for presenting the differences between the original samples), *χ*^2^ tests and *t* tests (Satterthwaite) were performed for categorical and continuous variables, respectively. Furthermore, loess regression was performed to visualize the unadjusted relationship between age (years) and binary complication rates.

## Results

Out of the 3209 patients with PEH repair, 381 (11.9%) were aged ≥ 80 years. The vast majority of the repairs were done laparoscopically in both groups, at 93.8% (< 80 years) and 91.4% (≥ 80 years), respectively.

### Unadjusted analysis before matching

When comparing the frequency distribution of the different matching variables, significant differences were found. The BMI in patients ≥ 80 years old was significantly lower compared to the BMI of younger patients (mean 26.6 ± 4.5 vs. 29.0 ± 5.0; *p* < 0.001). Patients ≥ 80 years had significantly fewer fundoplications, larger hernias, a higher ASA score, more risk factors, and were predominately female (Table 1).

**Table 1:** Unadjusted analysis for the matching variables between the two age groups

	Age				*p*
	< 80 Years		≥ 80 Years		
	*n*	%	*n*	%	
Fundoplication					
Fundophrenicopexy	517	18.28	113	29.66	< 0.001
Nissen fundoplication	973	34.41	110	28.87	
Toupet fundoplication	1044	36.92	116	30.45	
Other	294	10.40	42	11.02	
Access					
Laparoscopy	2653	93.81	348	91.34	0.075
Open	175	6.19	33	8.66	
Type of hernia					
Mixed	726	25.67	55	14.44	< 0.001
Paraesophageal	845	29.88	74	19.42	
Up-side-down stomach	1257	44.45	252	66.14	
Hiatal repair					
Other	29	1.03	5	1.31	0.138
Suture only	1793	63.40	220	57.74	
Suture and mesh	967	34.19	152	39.90	
Mesh	39	1.38	4	1.05	
ASA					
I	291	10.29	8	2.10	< 0.001
II	1654	58.49	120	31.50	
III/IV	883	31.22	253	66.40	
Sex					
Male	947	33.49	86	22.57	< 0.001
Female	1881	66.51	295	77.43	
Risk factors					
Overall					
Yes	904	31.97	166	43.57	< 0.001
No	1924	68.03	215	56.43	
COPD					
Yes	384	13.58	81	21.26	< 0.001
No	2444	86.42	300	78.74	
Diabetes mellitus					
Yes	204	7.21	41	10.76	0.018
No	2624	92.79	340	89.24	
Aortic aneurysm					
Yes	16	0.57	3	0.79	0.486
No	2812	99.43	378	99.21	
Immunosuppression					
Yes	33	1.17	4	1.05	1.000
No	2795	98.83	377	98.95	
Steroids					
Yes	62	2.19	15	3.94	0.048
No	2766	97.81	366	96.06	
Smoking					
Yes	191	6.75	5	1.31	< 0.001
No	2637	93.25	376	98.69	
Coagulation disorder					
Yes	48	1.70	15	3.94	0.009
No	2780	98.30	366	96.06	
Antiplatelet therapy					
Yes	224	7.92	72	18.90	< 0.001
No	2604	92.08	309	81.10	
Anticoagulation					
Yes	47	1.66	18	4.72	< 0.001
No	2781	98.34	363	95.28	

*ASA* American Society of Anesthesiologists status, *COPD* chronic obstructive pulmonary disease

### Standardized differences after propensity score matching

**Fig. 2:**
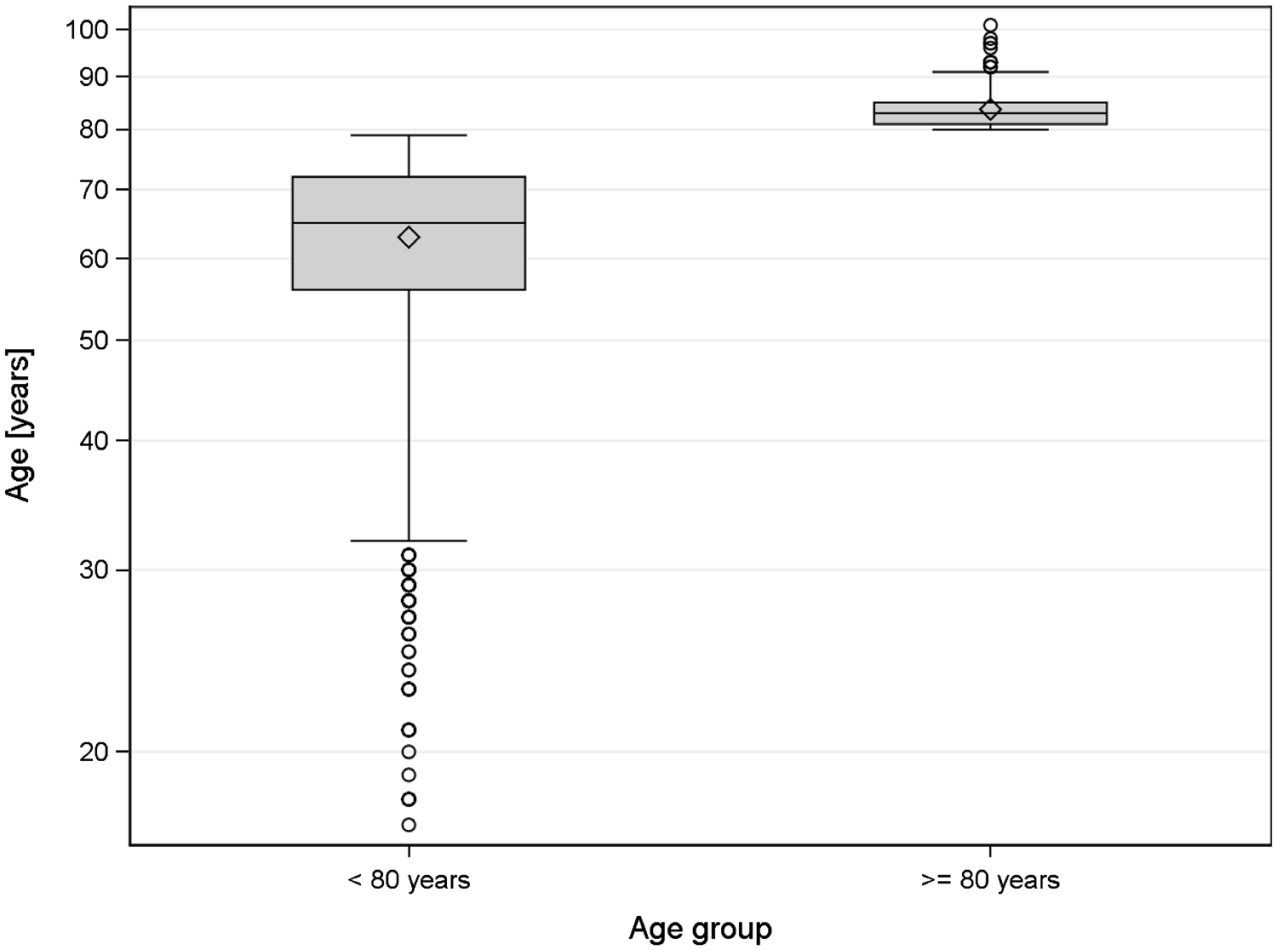
Age distribution within the age groups (after matching)

Matching was successfully applied for 360 patients ≥ 80 years (94.5%). The group < 80 years had a mean age of 68.2 years (SD 9.67), whereas the group ≥ 80 years had a mean age of 83.6 years (SD 3.21) (Fig. 2).

Table 2 shows the distribution after matching and the standardized differences in the categorical matching variables before (original sample) and after matching (matched sample). All the matching variables show a difference of less than 10%, providing a good balance of those variables in the matched sample. This also holds for BMI, which is 27.0 ± 4.4 and 26.8 ± 4.5 in patients < 80 years and patients ≥ 80 years after matching, respectively (standardized difference = 0.043).

**Table 2:** Standardized differences of the categorical matching parameters before and after matching

	< 80 Years		≥ 80 Years		Standardized difference	
	*n*	%	*n*	%	Matched sample	Original sample
Male	83	23.06	83	23.06	0.000	0.245
ASA I	6	1.67	8	2.22	0.040	0.345
ASA II	115	31.94	119	33.06	0.024	0.564
ASA III–IV	239	66.39	233	64.72	0.035	0.752
Other fundoplication	38	10.56	38	10.56	0.000	0.020
Nissen fundoplication	105	29.17	105	29.17	0.000	0.119
Toupet fundoplication	110	30.56	110	30.56	0.000	0.137
Fundophrenicopexy	107	29.72	107	29.72	0.000	0.269
Paraoesophageal	82	22.78	72	20.00	0.068	0.244
Mixed	46	12.78	54	15.00	0.064	0.283
Up-side-down stomach	232	64.44	234	65.00	0.012	0.447
Risk factors	168	46.67	154	42.78	0.078	0.241
Risk factor: COPD	79	21.94	77	21.39	0.013	0.204
Risk factor: diabetes mellitus	42	11.67	38	10.56	0.035	0.124
Risk factor: aortic aneurysm	2	0.56	3	0.83	0.033	0.027
Risk factor: immunosuppression	3	0.83	4	1.11	0.028	0.011
Risk factor: steroids	13	3.61	14	3.89	0.015	0.101
Risk factor: smoking	6	1.67	5	1.39	0.023	0.279
Risk factor: coagulation disorder	8	2.22	12	3.06	0.052	0.136
Risk factor: antiplatelet therapy	69	19.17	65	18.06	0.029	0.326
Risk factor: anticoagulation	13	3.61	14	3.89	0.015	0.175

*ASA* American Society of Anesthesiologists status, *COPD* chronic obstructive pulmonary disease

### Matched-pair analysis

**Fig. 3:**
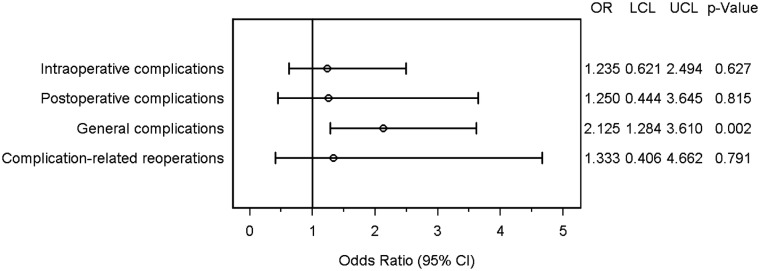
Forest plot—adjusted odds ratios. *OR* odds ratio, *LCL* lower confidence limit, *UCL* upper confidence limit

The matched-pair analysis revealed no systematic differences for intraoperative complications. There were 5.8% events only in the older group compared to 4.7% events only in younger patients (OR 1.235 [0.621; 2.494]; *p* = 0.627). Postoperative complications occurred in 2.8% of the matched pairs in older patients only and in 2.2% in younger patients only (OR 1.250 [0.444; 3.645]; *p* = 0.815). Patients ≥ 80 years showed significantly more general complications compared to the matched patients of the younger group (OR 2.125 [1.284; 3.610]; *p* = 0.002) (Fig. 3). On analyzing the frequency distribution of single general complications, only pneumonia showed a significant difference between the two groups (*p* = 0.041). There was no systematic difference in mortality (OR 2.000 [0.215; 35.199]; *p* = 1.000) or in any of the other general complications between the two groups (Table 3). Finally, no systematic differences were found between age groups for complication-related reoperations (1.7% vs.·2.2%, OR = 1.333 [0.406; 4.662], *p* = 0.791).

**Table 3:** General complications

	Disadvantage		*p*-value*	OR* for matched samples		
	< 80 Years	≥ 80 Years		OR	Lower limit	Upper limit
Fever	1.11	1.11	1.000	1.000	0.079	12.702
Urinary tract infection	0.83	2.22	1.000	2.667	0.351	44.213
Diarrhea	0.56	0.56	1.000	1.000	0.017	60.294
Gastritis	0.00	0.00				
Thrombosis	0.00	0.00				
Pulmonary embolism	0.83	0.28	1.000	0.333	0.000	12.420
Pleural effusion	2.50	4.44	1.000	1.778	0.497	7.451
Pneumonia	0.83	4.72	0.041	5.667	1.028	84.669
COPD	1.11	1.39	1.000	1.250	0.127	14.796
Heart failure	0.83	2.78	1.000	3.333	0.495	53.213
Coronary heart disease	0.56	1.11	1.000	2.000	0.118	95.634
Myocardial infarction	0.28	0.56	1.000	2.000	0.024	1918.000
Renal failure	0.83	1.11	1.000	1.333	0.094	26.159
Hypertensive crisis	0.56	0.83	1.000	1.500	0.059	77.990
Death	0.83	1.67	1.000	2.000	0.215	35.199
Other complications	1.11	3.89	0.494	3.500	0.686	33.477

Relative frequency of cases with disadvantage for the respective age group (non-diagonal elements of 2 × 2 contingency table)

*OR* odds ratio, *COPD* chronic obstructive pulmonary disease

*Adjusted according to Bonferroni: factor 16

### Loess regression

**Fig. 4:**
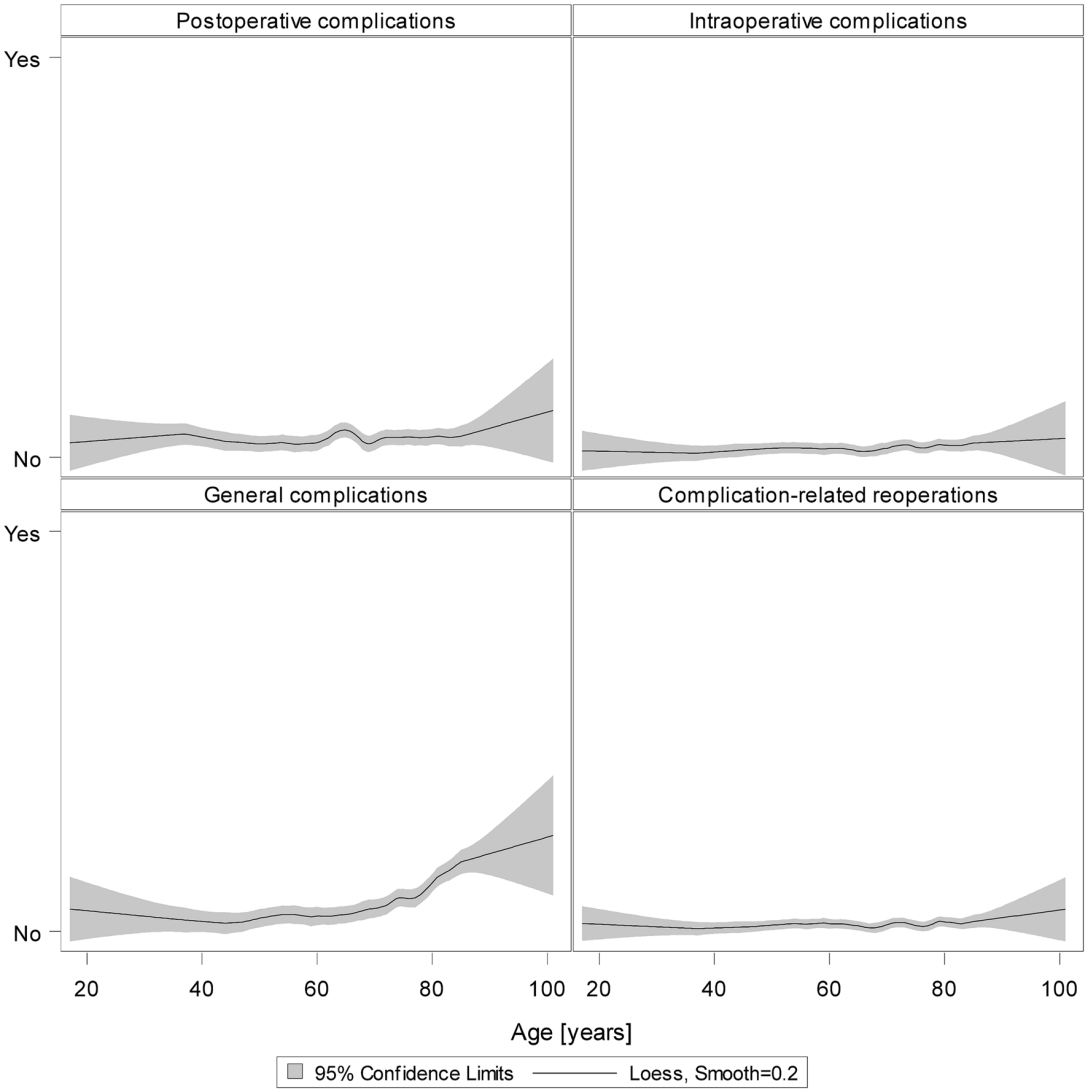
Loess regression for postoperative, intraoperative, and general complications as well as complication-related reoperations for all patients (smooth = 0.2)

The results of unadjusted loess regression on all 3209 patients underline our results: Except for general complications, there were no reliable signs that more complications occurred in the older group (Fig. 4).

**Fig. 5:**
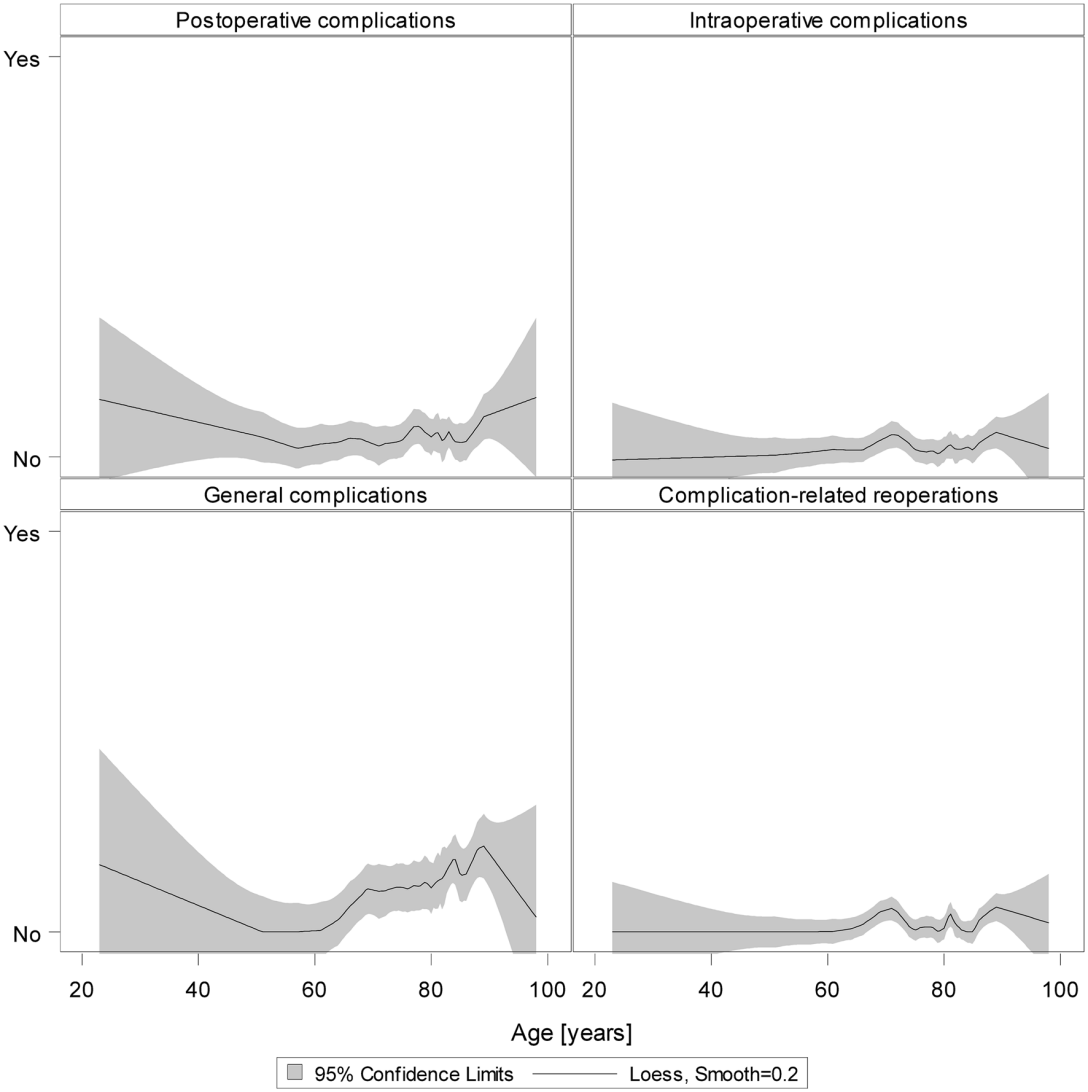
Loess regression for postoperative, intraoperative and general complications as well as complication-related reoperations for patients of the matched sample (smooth = 0.2)

Furthermore, the results of unadjusted loess regression on only those patients of the matched samples revealed that the controls (patients < 80 years) who were chosen for matching because of their comparable characteristics did not show higher complication rates in higher ages only (Fig. 5).

### Unadjusted analysis of 1-year follow-up data

If we restrict the analysis population to those patients with one-year follow-up data, *n* = 1505 patients < 80 years old (53.2%) and *n* = 160 patients ≥ 80 years old (42.0%) remain. Since these follow-up rates are profoundly different, one can assume that patient inclusion is strongly biased, e.g., restricted to those patients ≥ 80 years old who are relatively healthy. Nevertheless, we provide the recurrence rate, which is 4.8% in patients < 80 years old (*n* = 72) and 1.9% in patients ≥ 80 years old (*n* = 3), respectively (*p* = 0.092).

## Discussion

This is the first propensity score-based, matched-pair analysis evaluating the complication rates of elective PEH in patients ≥ 80 years old. Our study showed that elderly patients can undergo PEH with intraoperative and postoperative surgical complication rates comparable with those of younger patients. The only general complication that was significantly more frequent after PEH in patients ≥ 80 years was pneumonia, highlighting the postoperative respiratory vulnerability of this patient population.

This study contributes to the ongoing and important discussion of balancing the perioperative risks and the supposed postoperative benefit of surgical procedures in elderly patients. Due to demographic trends in most countries, surgical patients increasingly present at an advanced age and with more comorbidities. It is accepted that advanced age in itself does not increase perioperative morbidity and mortality, and therefore there is no age limit for surgical interventions [15]. However, making therapeutic decisions for or against surgical treatment seems more challenging in older patients, since comorbidities may increase the surgical risk. Regarding PEH repair, one can argue that elective surgical treatment is the method of choice since symptoms may not be controlled with conservative treatment strategies and prevention of emergency situations with significantly higher morbidity and mortality seems appropriate [6]. There is a paucity of high-level evidence literature on elective PEH repair in elderly patients. A few studies defined elderly as > 70 years [3, 8, 9] or analyzed a very small group of elderly patients [5, 7, 10, 16], making comparison with our data difficult. Only one study evaluating elective PEH repair in 313 patients ≥ 80 years revealed a significant increase in minor morbidity (8.3% vs. 3.5%, *p* < 0.001), and a trend towards slightly higher mortality (1% vs. 0.4%, *p* = 0.16) and major morbidity (5.8% vs. 3.7%, *p* = 0.083) for patients ≥ 80 years [11]. The authors concluded that PEH repair can be performed with minimal morbidity and mortality in elderly patients. However, the main limitation of this study and most other observational studies is its confounding bias, especially when comparing two very different and unequal patient populations. Our propensity score, registry-based study revealed comparable rates for perioperative and postoperative surgical complications for elderly and younger patients, underlining the safety of the surgical approach itself in the older patient population.

Our findings may have some clinical impact. Since the natural course of untreated PEH is estimated to be associated with an annual symptom progression in 14% of patients, requiring emergency surgery in 1.1% of cases [17, 18], elective surgery seems important, especially for elderly patients. Our data support the concept of elective PEH repair in elderly patients with a low surgical mortality and morbidity. The surgical approach in elderly patients with PEH seems appropriate to significantly improve the quality of life [3] and prevent higher mortality and morbidity rates in emergency settings [6, 19, 20]. However, the higher rate of postoperative pneumonia in the older patient population underlines the importance of careful perioperative management and preventive strategies for general complications. Perioperative physiotherapy and respiratory training may help to reduce the risk of pulmonary complications after surgery [21].

Since this is a registry-based study, there are some limitations. Data on preventive respiratory strategies such as breathing exercises or inhalations are not recorded in the Herniamed Registry. Therefore, the potential effect of preventive respiratory physiotherapy in our patient population remains unknown. However, the following measurements are used to optimize data entry in the Herniamed Registry: signed contract with the responsible surgeon for data correctness and completeness, indication of missing data by the software, once again review of the perioperative outcome at 1-year follow-up and control of the data entry by experts as part of the certification process of hernia centers. Furthermore, to overcome the confounding bias of analyzing two different patient populations, a propensity score (PS) was applied in our study [22].

In summary, our study shows that age ≥ 80 years in itself is not a risk factor for higher intraoperative or postoperative complication rates compared to younger patients in elective PEH repair. However, careful perioperative management with prevention of respiratory complications seems of utmost importance in elderly patients. Further studies investigating recurrence rates and long-term complications are needed to evaluate the effectiveness of elective PEH repair in octogenarians and nonagenarians.

## Electronic supplementary material

Below is the link to the electronic supplementary material.

Supplementary material 1 (DOCX 19 KB)
